# The Relationship Between Metabolic Syndrome and Pain Catastrophizing in Psoriatic Arthritis

**DOI:** 10.1007/s40744-025-00758-6

**Published:** 2025-03-26

**Authors:** Damiano Currado, Onorina Berardicurti, Francesca Saracino, Francesca Trunfio, Lyubomyra Kun, Annalisa Marino, Erika Corberi, Ludovica Lamberti, Piero Ruscitti, Vasiliki Liakouli, Marta Vadacca, Amelia Rigon, Luisa Arcarese, Manuela Pietramale, Francesco De Vincenzo, Marta Vomero, Francesco Ciccia, Roberto Giacomelli, Luca Navarini

**Affiliations:** 1 Unit of Allergology, Clinical Immunology and Rheumatology, Department of Medicine, University Campus Bio-Medico di Roma, via Alvaro del Portillo 21, 00128 Rome, Italy; 2 Clinical and Research Section of Rheumatology and Clinical Immunology, Fondazione Policlinico Campus Biomedico, Rome, Italy; 3 Department of Biotechnological and Applied Clinical Sciences, University of L’Aquila, L’Aquila, Italy; 4 Rheumatology Unit, Department of Precision Medicine, University of Campania L. Vanvitelli, Caserta, Italy; 5 Department of Human Sciences, European University of Rome, Rome, Italy

**Keywords:** Metabolic syndrome, Pain catastrophizing, Psoriatic arthritis

## Abstract

**Introduction:**

Psoriatic arthritis (PsA) is a complex inflammatory disease often associated with metabolic syndrome (MetS). It has been demonstrated that pain catastrophizing (PC), characterized by an exaggerated negative cognitive and emotional response to actual or anticipated pain, impacts the achievement of remission and therapy discontinuation in patients with PsA. In this study, we evaluate the potential role of MetS, the most prevalent comorbidity in PsA, in influencing PC in patients with PsA.

**Methods:**

We conducted a cross-sectional, observational study on 170 patients with PsA who met the Classification Criteria for PsA and MetS criteria. Data on disease activity, PC, and comorbidities were collected and analyzed using univariable and multivariable regressions.

**Results:**

Our results indicate a significant association between MetS and elevated PC levels in patients with PsA. Univariable analysis identified female gender, fibromyalgia, and higher Disease Activity for Psoriatic Arthritis (DAPSA) scores as factors associated with increased PC. Multivariable analysis, adjusted for age, sex, fibromyalgia, and DAPSA, confirmed that MetS independently correlates with higher PC levels (*b* = 8.84, 95% CI 4.66–13.02, *p* < 0.0001) and its domains (helplessness, rumination, magnification).

**Conclusions:**

These findings suggest that MetS significantly impacts PC in PsA, underscoring the need for a multidisciplinary approach to patient management. This study highlights the importance of addressing MetS to reduce pain catastrophizing and enhance disease management in PsA.

## Key Summary Points

**Table:** 

***Why carry out this study?***
Metabolic syndrome (MetS) is a common comorbidity in psoriatic arthritis (PsA), associated with increased cardiovascular risk and worse pain perception.
Psoriasis and PsA share inflammatory mechanisms and metabolic abnormalities, but the role of pain catastrophizing (PC) in patients with psoriatic arthritis and MetS has not been previously investigated.
This study aimed to examine the relationship between MetS and PC in patients with PsA, hypothesizing a significant association between MetS and higher levels of PC.
***What was learned from the study?***
MetS was strongly associated with higher levels of PC in patients with psoriatic arthritis, both in overall PC scores and across its components (helplessness, rumination, and magnification).
The presence of MetS negatively impacts the management of psoriatic arthritis, reducing the likelihood of achieving remission or low disease activity.
These findings emphasize the need for a multidisciplinary approach to improve metabolic health and psychological well-being in patients with psoriatic arthritis.

## Introduction

Psoriatic arthritis (PsA) is a complex inflammatory disease that occurs in patients affected by or with a family history of psoriasis (PsO). PsA has heterogeneous clinical features, affecting axial and /or peripheric joints and enthesis [1]. PsA often presents alongside several comorbidities, one of which is metabolic syndrome (MetS). MetS is characterized by a combination of hyperglycemia/insulin resistance, obesity, hypertension, and dyslipidemia, conditions that collectively increase the risk for cardiovascular diseases. It is well known that in PsA and other inflammatory arthritides, patients exhibit an elevated cardiovascular risk, partly due to systemic inflammation, which a comorbid MetS can further exacerbate [2–6].

PsA and Mets share common inflammatory mechanisms and metabolic abnormalities. In particular, the role of proinflammatory cytokines, such as tumor necrosis factor (TNF)-α, interleukin (IL)-6, and IL-17 is known to be crucial in both conditions. Furthermore, chronic inflammation can lead to insulin resistance, which plays a key role in MetS development [2]. Insulin resistance may further exacerbate inflammation, thus creating a vicious loop. It is also known that adipocytes release proinflammatory cytokines (adipokines) such as IL-6, TNF-α, and leptin, contributing to systemic inflammation of PsA [7, 8]. All these inflammatory pathways can lead to increased oxidative stress, exacerbating endothelial damage, which is a primary contributor to cardiovascular diseases. Finally, alterations in gut microbiota have been observed to be associated with both PsA and MetS; dysbiotic gut microbiota can promote systemic inflammation and metabolic dysfunction [2].

Recent reports indicate a link between obesity and pain perception, leading to a reduction in quality of life, especially for patients with chronic conditions [9–12]. Still, other MetS features, such as insulin resistance [13–15], hypertension [16], and dyslipidemia [17, 18], seem to be related to an altered nociception.

On the other hand, pain in inflammatory arthritides depends on joint inflammation, but it is also strongly associated with a broad spectrum of psychological factors: among them, pain catastrophizing (PC) seems to play a crucial role.

The concept of catastrophizing was initially introduced by Albert Ellis and then adapted by Aaron Beck to describe a maladaptive cognitive style in patients with anxiety and depressive disorders. Catastrophization exaggerates negative cognitive and emotional responses during actual or anticipating painful situations [19–23].

PC, as measured by the Pain Catastrophizing Scale (PCS), includes three components: the inability to suppress pain-related thoughts (i.e., rumination), the exaggeration of the threat value of pain (i.e., magnification), and the tendency to feel helpless about one’s ability to cope with painful situations [24, 25].

In previous studies, our and other groups showed that PC negatively impacts the achievement of remission and/or low disease activity in inflammatory arthritis. Furthermore, we found a significant relationship between an increased PC, as a whole and for each domain, and therapy discontinuation in patients with PsA [26–29].

To our knowledge, the association between PC and MetS in PsA has never been investigated. The present study aims to examine the relationship between MetS and PC.

## Methods

A single-center, observational study has been conducted on PsA participants enrolled in our Arthritis Center, Rheumatology Clinic of Fondazione Policlinico Campus Bio-Medico of Rome. Consecutive outpatients have been enrolled from November 2023 to June 2024; at baseline, PsA participants fulfilled the Classification Criteria for Psoriatic Arthritis (CASPAR) [30]. The study was approved by the Ethics Committee of the University Campus Bio-Medico of Rome and conducted in conformity with the Declaration of Helsinki and its later amendments (approval no. 78.20 OSS). Written informed consent was obtained from all enrolled patients.

Participants fulfilling the US National Cholesterol Education Program Adult Treatment Panel III (NCEP-ACT III) criteria were classified as MeTs [31].

Inclusion criteria were: both genders, age > 18 years and the fulfilment of CASPAR criteria. The exclusion criteria were: history of any malignancy, pregnancy, age > 85, inability to express informed consent to participate in the study, and history of any psychiatric disorder according to DSM-V before the recruitment.

At baseline, the following PsA disease activity scores were collected by clinicians who carried out the visit: Disease Activity for Psoriatic Arthritis (DAPSA), minimal disease activity (MDA), very low disease activity (VLDA), Bath Ankylosing Spondylitis Disease Activity Index (BASDAI), Psoriasis Area Severity Index (PASI), Leeds Enthesitis Index (LEI). For DAPSA, we summed the following variables: tender and swollen joints count (TJC68, SJC66), patient global assessment (PtGA), patient pain on a 10-cm visual analogue scale (VAS), and C-reactive protein (CRP). Axial involvement was assessed based on the patient's clinical history, where MRI or X-ray findings of the sacroiliac joints or spine were considered positive for psoriatic arthritis axial involvement. The presence of psoriasis at the time of the visit or a history of psoriasis (reported in the patient's medical history) was recorded.

Pain catastrophizing, with its domains of helplessness, rumination and magnification, was analyzed through PCS, which takes into account the three domains of pain catastrophizing. The first component, "rumination," comprises four items related to ruminative thoughts, worry, and the inability to suppress pain-related thoughts. The second component, "magnification," consists of three items reflecting the exaggeration of pain's unpleasantness and the anticipation of negative outcomes. The third component, "helplessness," encompasses five items from the Coping Strategies Questionnaire (CSQ) and an additional item indicating the inability to cope with painful situations. Health Assessment Questionnaire (HAQ) for the disability and the physical function, Hospital Anxiety and Depression Scale (HADS) for the depressive-anxious symptoms, Trait Hope Scale (THS) for hope, Acceptance and Action Questionnaire (AAQ) for experiential avoidance were analyzed via standardized questionnaires.

Continuous variables are described as median (25–75th percentile), whilst categorical variables are described as percentages (%). The Shapiro–Wilk test was used to evaluate the normality of data. χ^2^ was used to analyze contingency tables, while Mann–Whitney test was used to compare ranks. To evaluate variables associated with pain catastrophizing and its domains, univariable and multivariable regressions were used, considering for the multivariable analysis every variable with *p* < 0.1 in the univariate analysis plus age and sex. The whole statistical analysis was performed using Stata v.14 and *p* values < 0.05 have been considered significant.

## Results

The main demographic and clinical characteristics of the study population are reported in Table 1.

**Table 1:** Main demographic, anthropometric, and clinical characteristics of the study population

	Entire PsA participants 170	PsA participants without MeTs, 97 (57.06%)	PsA participants with MeTs, 73 (42.94%)	*p* value (PsA participants with MeTs vs. without MetS)
Age (median 25–75% percentile)	58 (53–64)	56 (51–63)	60 (54–65)	0.005
Sex, female, *n* (%)	113 (66.4%)	59 (60.8%)	54 (73.9%)	0.007
Disease duration, months	72 (25–132)	61 (24.5–120)	79 (27–150)	0.2
Weight, kg	75 (66–85)	70 (63–81)	80 (73–89)	< 0.0001
BMI, kg/m^2^	27 (24.5–30)	26 (23.5–28.4)	29.1 (26.6–32.82)	< 0.0001
Waist circumference, cm	91 (81–102)	85 (75–95.5)	100 (93–106)	< 0.0001
Triglycerides, mg/dl	100 (80–149)	90 (80–109)	143 (91–172)	< 0.0001
HDL, mg/dl	56 (50–65)	60 (54–66)	50 (43–56)	< 0.0001
Hypertension or therapy	84 (52.17%)	25 (27.78%)	59 (83.1%)	< 0.0001
Blood glucose, mg/dl	90 (86–103)	90 (85–96)	103 (89–118)	< 0.0001
Fibromyalgia	63 (37.5%)	23 (23.71%)	40 (56.34%)	< 0.0001
Smokers	61 (35.8)	28 (28.87)	33 (45.21)	0.02
*Peripheral joint*				
Involvement				0.6
Oligoarticular	128 (77.11%)	73 (76.04%)	55 (78.57%)	
Polyarticular	25 (15.06%)	14 (14.58%)	11 (15.71%)	
Axial involvement	81 (50%)	45 (48.39%)	36 (52.17%)	0.6
Enthesitis	68 (40%)	41 (42.27%)	27 (36.99%)	0.4
Psoriasis	103 (61.3%)	64 (65.9%)	39 (54.9)	0.1
Onicopsoriasis	33 (19.6%)	17 (17.53)	16 (22.54)	0.4
Uveitis	3 (1.79%)	2 (1.08%)	1 (1.39%)	0.7
IBD	10 (5.99%)	7 (7.29%)	3 (4.23%)	0.4
*cDMARDs*				
Methotrexate	46 (27.06%)	29 (29.9%)	17 (23.29%)	0.5
Salazopirin	13 (7.65%)	5 (5.15%)	8 (10.96%)	
Leflunomid	10 (5.88%)	4 (4.12%)	6 (8.22%)	
Cyclosporine	2 (1.17%)	1 (1.03%)	1 (1.37%)	
Hydroxychloroquine	2 (1.17%)	1 (1.03%)	1 (1.37%)	
*bDMARDs*				
Infliximab	2 (1.17%)	1 (1.03%)	1 (1.37%)	0.3
Adalimumab	56 (32.94%)	35 (36.08%)	21 (28.77%)	
Etanercept	15 (8.82%)	8 (8.25%)	7 (9.59%)	
Golimumab	13 (7.65%)	6 (6.19)	7 (9.59%)	
Certolizumab	1 (0.59)	0	1 (1.37%)	
Secukinumab	13 (7.65%)	7 (7.22%)	6 (8.22%)	
Ixekizumab	1 (0.59)	0	1 (1.37%)	
Ustekinumab	11 (6.47%)	4 (4.12%)	7 (9.59%)	
Apremilast	7 (4.12%)	2 (2.06)	5 (6.85)	
Risankizumab	2 (1.18%)	1 (1.03%)	1 (1.37%)	
Guselkumab	3 (1.76%)	1 (1.03)	2 (2.74%)	
CCS (*n*%)	27 (15.8%)	17 (17.53%)	10 (13.7%)	0.5
NSAIDs (*n*%)	46 (27.22%)	23 (23.71%)	23 (31.94%)	0.2
TJ	1 (0–5)	1 (0–4)	2 (0–7)	0.02
SJ	0 (0–1)	0 (0–0)	0 (0–2)	0.003
PP	5 (3–8)	4 (1–7)	7 (5–8)	< 0.0001
PtGA	5 (2–7)	4 (1–7)	6 (3–8)	0.0003
EGA	0 (0–2)	0 (0–1)	1 (0–2)	0.003
CRP mg/dl	0.26 (0.1–0.45)	0.26 (0.1–0.44)	0.3 (0.1–0.48)	0.7
ESR, mm/h	14 (7–21)	13 (7–19)	16 (8–28)	0.04
HAQ	1 (0.1–1.63)	0.5 (0–1.25)	1.63 (0.75–2)	< 0.0001
DAPSA	12.5 (4.07–22)	8.4 (3.3–17.3)	19.25 (10.1–23.1)	0.0001
BASDAI	5.1 (2.4–7.1)	3.8 (1.8–6.8)	6.35 (4.3–7.9)	0.0002
MDA	62 (37.58%)	48 (50.53%)	14 (20%)	< 0.0001
VLDA	29 (17.37%)	25 (26.04%)	4 (5.63)	0.001
PCS	18 (6–32)	9 (5–20)	30 (18–40)	< 0.0001
Helplessness	7 (2–14)	4 (1–9)	12 (7–16)	< 0.0001
Rumination	7 (2–13.5)	5 (1–10)	13 (7–16)	< 0.0001
Magnification	3 (1–5)	1 (0–4)	5 (2–7)	< 0.0001
HADS anxiety	6 (3–10)	5 (2–8)	9 (3–13)	0.001
HADS depression	5 (2–8)	4 (1–7)	6 (4–10)	0.0006
AAQ-II	15 (9–25.5)	12 (8–20)	23 (11–32)	0.001
Trait Hope Scale	25 (21–27)	25 (22–28)	24 (21–26)	0.06

*PsA* psoriatic arthritis, *MetS* metabolic syndrome, *BMI* body mass index, *HDL* high-density-lipoprotein, *IBD* inflammatory bowel disease, *csDMARDs* conventional synthetic disease-modifying anti-rheumatic drugs, *bDMARDs* biological disease-modifying anti-rheumatic drugs, *CCS* corticosteroids, *NSAIDs* nonsteroidal anti-inflammatory drugs, *TJ* tender joints, *SJ* swollen joints, *PP* patient pain, *PtGA* patients’ global assessment, *EGA* physician global assessment, *CRP* C-reactive protein, *ESR* erythrocyte sedimentation rate, *HAQ* Health Assessment Questionnaire, *DAPSA* Disease Activity for Psoriatic Arthritis, *MDA* minimal disease activity, *VLDA* activity very low disease, *BASDAI* Bath Ankylosing Spondylitis Disease Activity Index, *PCS* Pain Catastrophizing Scale, *HADS* Hospital Anxiety and Depression Scale, *AAQ* Acceptance and Action Questionnaire

Conventional synthetic disease-modifying anti-rheumatic drugs (csDMARDs) were used by 42.93% of the patients, and particularly, 27.06% were receiving methotrexate, 7.65% sulfasalazine, 1.17% cyclosporine, 1.17% hydroxychloroquine, and 5.88% leflunomide. Biologic DMARDs (bDMARDs) or the phosphodiesterase-4 (PDE-4) inhibitor, were used by 72.94% of the patients and particularly, 1.17% infliximab, 8.82% etanercept, 32.94% adalimumab, 0.59% certolizumab pegol, 7.65% golimumab, 6.47% ustekinumab, 7.65% secukinumab, 0.59% ixekizumab, 1.18% risankizumab, 1.76% guselkumab and 4.12% apremilast. According to NCEP-ACT III criteria, 42.94% of patients were classified as having MetS [31].

Our cohort showed a median DAPSA value of 12.5 (4.07–22) and a median BASDAI score of 5.1 (2.4–7.1). 62 (37.58%) participants achieved MDA criteria, and VLDA was achieved only in 29 (17.37%) participants. Concomitant fibromyalgia was present in 63 (37.5%) participants.

We observed a PCS median value of 18 (6–32). Regarding its domains, the Helplessness median value was 7 (2–14), the Rumination median value was 7 (2–13.5), and the Magnification median value was 3 (1–5).

To evaluate the relationship between PC and MeTs, univariable and multivariable linear regressions were performed, and the results are reported in Tables 2 and 3, respectively.

**Table 2:** Univariable regression (PCS as dependent variable)

PCS (dependent variable)	Coeff. b	95% CI	*p* value
Female sex	8.61	4.02 to 13.2	< 0.0001
BMI	0.64	0.15 to 1.13	0.01
Hypertension	6.45	1.96 to 10.94	0.005
Blood glucose	0.11	– 0.004 to 0.21	0.05
Metabolic syndrome	13.96	9.94 to 17.98	< 0.001
Fibromyalgia	12.24	8 to 16.48	< 0.0001
TJ	1.03	0.52 to 1.54	< 0.0001
SJ	2.2	0.75 to 3.65	0.003
PP	2.83	2.22 to 3.45	< 0.0001
PtGA	2.67	2.04 to 3.3	< 0.0001
EGA	3.61	1.72 to 5.5	< 0.0001
HAQ	10.71	8.55 to 12.87	< 0.0001
DAPSA	0.78	0.58 to 0.97	< 0.0001
BASDAI	3.38	2.7 to 4.05	< 0.0001
MDA	– 17.29	– 21.14 to – 13.44	< 0.0001
VLDA	– 17.57	– 22.86 to – 12.27	< 0.0001

*PCS* Pain Catastrophizing Scale, *BMI* body mass index, *TJ* tender joints, *SJ* swollen joints, *PP* patient pain, *PtGA* patients’ global assessment, *EGA* physician global assessment, *HAQ* Health Assessment Questionnaire, *DAPSA* Disease Activity for Psoriatic Arthritis, *BASDAI* Bath Ankylosing Spondylitis Disease Activity Index, *MDA* minimal disease activity, *VLDA* very low disease activity

**Table 3:** Multivariable regression; PCS, helplessness, rumination and magnification, as dependent variables; model adjusted for age, sex, fibromyalgia, DAPSA (independent variables)

	Coeff. b	95% CI	*p* value
PCS as dependent variable			
Age	– 1.14	– 0.30 to 0.26	0.09
Sex	1.11	– 3.19 to 5.40	0.6
Mets	8.84	4.66 to 13.02	< 0.0001
Fibromyalgia	3.60	– 1.07 to 8.27	0.1
DAPSA	0.55	0.34 to 0.77	< 0.0001
Helplessness as dependent variable			
Age	– 0.6	– 0.13 to 0.1	0.09
Sex	0.16	– 0.17 to 2.03	0.8
Mets	3.67	1.85 to 5.49	< 0.0001
Fibromyalgia	1.99	– 0.37 to 4.03	0.05
DAPSA	0.25	0.16 to 0.35	< 0.0001
Rumination as dependent variable			
Age	– 0.70	– 0.15 to 0.003	0.06
Sex	0.3	– 1.62 to 2.21	0.7
Mets	3.93	2.07 to 5.79	< 0.0001
Fibromyalgia	0.58	– 1.5 to 2.67	0.5
DAPSA	0.25	0.16 to 0.35	< 0.0001
Magnification as dependent variable			
Age	– 0.01	– 0.05 to 0.02	0.6
Sex	0.54	– 0.4 to 1.5	0.2
Mets	1.42	0.5 to 2.35	0.003
Fibromyalgia	1.07	0.4 to 2.1	0.004
DAPSA	0.05	– 0.001 to 0.09	0.05

*PCS* Pain Catastrophizing Scale; *MetS* metabolic syndrome; *DAPSA* Disease Activity for Psoriatic Arthritis

Of note, the adjusted univariable regression model showed a positive association between PCS and female sex (*b* = 8.61, 95% CI 4.02 to 13.2, *p* < 0.001), DAPSA values (*b* = 0.78, 95% CI 0.58 to 0.97, *p* < 0.001), diagnosis of fibromyalgia (*b* = 12.24, 95% CI 8 to 16.48, *p* < 0.001), and presence of MetS (*b* = 13.96, 95% CI 9.94 to 17.98, *p* < 0.001).

Multivariable linear regression (adjusted for age, sex, fibromyalgia, and DAPSA disease activity) showed a significant association between PCS and Mets (*b* = 8.84, 95% CI 4.66–13.02, *p* < 0.0001), and between PCS and DAPSA (*b* = 0.55, 95% CI 0.34 to 0.77, *p* < 0.0001), as reported in Table 3.

Finally, multivariable linear regression (adjusted for age, sex, fibromyalgia, and DAPSA disease activity) showed a significant relationship between each of the three components of PCS (helplessness, rumination, and magnification) and MetS as well as DASPA scores, as reported in Table 3. The same significant associations were observed in multivariable linear regression using MDA rather than DAPSA as a measure of disease activity, as shown in Table 4.

**Table 4:** Multivariable regression; PCS, helplessness, rumination and magnification, as dependent variables; model adjusted for age, sex, fibromyalgia, MDA (independent variables)

	Coeff. b	95% CI	*p* value
PCS as dependent variable			
Age	– 0.17	– 0.33 to – 0.1	0.036
Sex	2.1	– 2.05 to 6.24	0.3
Mets	9.74	5.85 to 13.63	< 0.0001
Fibromyalgia	2.33	– 1.94 to 6.61	0.28
MDA	– 12.92	– 17.07 to – 8.76	< 0.0001
Helplessness as dependent variable			
Age	0.08	– 0.15 to – 0.01	0.028
Sex	0.77	– 1.09 to 2.61	0.42
Mets	4.45	2.72 to 6.19	< 0.0001
Fibromyalgia	1.42	– 0.49 to 3.33	0.14
MDA	– 5.37	– 7.22 to – 3.52	< 0.0001
Rumination as dependent variable			
Age	– 0.08	– 0.15 to – 0.02	0.017
Sex	0.48	– 1.32 to 2.27	0.6
Mets	3.75	2.07 to 5.43	< 0.0001
Fibromyalgia	0.3	– 1.54 to 2.15	0.74
MDA	– 6.53	– 8.32 to – 4.73	< 0.0001
Magnification as dependent variable			
Age	– 0.01	– 0.4 to 0.3	0.62
Sex	0.75	– 0.17 to 1.67	0.11
Mets	1.69	0.82 to 2.55	< 0.0001
Fibromyalgia	0.64	– 0.3 to 1.59	0.18
MDA	– 1.01	– 1.93 to – 0.09	0.032

*PCS* Pain Catastrophizing Scale, *MetS* metabolic syndrome, *MDA* minimal disease activity

## Discussion

In this study, we provide valuable insights into the interplay between MetS and PC in patients affected by PsA. Our findings indicate that MetS is strongly associated with PC in patients with PsA. This association has a clinical relevance, considering that MetS affected nearly 43% of our recruited participants with PsA, mirroring other results reported in the literature [2, 32, 33].

Even though the association between MetS and increased pain perception in patients experiencing chronic pain is already known in the literature [9, 34], to the best of our knowledge, our paper is the first work exploring the interrelationship between a measurable maladaptive response to chronic pain and MetS.

Our univariate analysis identified factors positively associated with higher PCS levels, such as female sex, fibromyalgia, and higher DAPSA scores. These findings are consistent with the well-known association between the female sex and higher PC levels and also with data showing that higher levels of PC are found in patients with fibromyalgia [21, 21, 35]. Furthermore, regarding the DAPSA score, we recently showed a positive association between PCS and DAPSA and between PCS and BASDAI. Thereby, PC negatively affects the likelihood of remission and/or low disease activity in PsA [26–28]. These associations should be considered in the clinical setting because they may influence the therapeutic process strategies. In a previous paper, we showed a significant relationship between higher levels of PC (as a whole and for each domain) and therapy discontinuation [36].

When adjusted for age, gender, fibromyalgia, and DAPSA, our multivariable analysis confirmed the independent association between higher PC levels and MetS. This strong association highlights the significance of MetS as a critical factor affecting PC as a whole, along with each component of this maladaptive cognitive impairment (helplessness, rumination, and magnification).

Available literature reports some association among PC, obesity, and other chronic conditions, such as osteoarthritis, cancer, or migraine [37, 38]. In our cohort, we observed that patients with MetS showed a significant increase in both waist circumference and BMI. Furthermore, a significant association between obesity and PC was observed. Thus, we may speculate that among different domains of MeTs, increased waist circumference and related obesity could play a critical role in determining PC, mirroring what has already been reported in patients with obesity affected by other chronic pain conditions. The mechanisms involved in the PC during obesity remain unclear. Both the release of specific pain-related inflammatory cytokines from inflamed adipose tissue and the altered self-perception associated with obesity have been considered, although robust data are still lacking [39].

Furthermore, we found that a significantly higher portion of patients with MetS also had comorbid fibromyalgia. This result confirms data concerning the strong association between MetS and fibromyalgia, already shown in previous studies [40–42].

We acknowledge certain limitations in our study, including the relatively small sample size, which may not fully capture the heterogeneity of the disease, and the cross-sectional design that prevents us from identifying potential changes in PC over time, possibly influenced by adjustments in DMARD therapy or psychological interventions. However, despite these limitations, the screening for prior psychological interventions in our participants allows us to establish a well-defined cohort for a more accurate assessment of the role of PC.

## Conclusions

The higher prevalence of MetS in patients with PsA and its association with a higher PC negatively impacting the achievement of disease remission or low disease activity [26] suggests that a multidisciplinary approach is strongly needed. Interventions targeting both metabolic health and psychological well-being, such as lifestyle modifications, pharmacological treatments, and cognitive-behavioral therapy, could potentially improve the management of patients with PsA affected by MetS.

## Data Availability

The datasets used and analyzed during the current study are available from the corresponding author on reasonable request.
